# Evaluating Computer Screen Time and Its Possible Link to Psychopathology in the Context of Age: A Cross-Sectional Study of Parents and Children

**DOI:** 10.1371/journal.pone.0140542

**Published:** 2015-11-04

**Authors:** Aviv Segev, Aviva Mimouni-Bloch, Sharon Ross, Zmira Silman, Hagai Maoz, Yuval Bloch

**Affiliations:** 1 Shalvata Mental Health Center, Hod Hasharon, Israel; 2 The Pediatric Neurology and developmental Unit, Loewenstein Rehabilitation Hospital, Raanana, Israel; 3 Sackler Faculty of Medicine, Tel Aviv University, Tel Aviv, Israel; 4 Independent researcher, Netanya, Israel; University of California, San Francisco, UNITED STATES

## Abstract

**Background:**

Several studies have suggested that high levels of computer use are linked to psychopathology. However, there is ambiguity about what should be considered normal or over-use of computers. Furthermore, the nature of the link between computer usage and psychopathology is controversial. The current study utilized the context of age to address these questions. Our hypothesis was that the context of age will be paramount for differentiating normal from excessive use, and that this context will allow a better understanding of the link to psychopathology.

**Methods:**

In a cross-sectional study, 185 parents and children aged 3–18 years were recruited in clinical and community settings. They were asked to fill out questionnaires regarding demographics, functional and academic variables, computer use as well as psychiatric screening questionnaires. Using a regression model, we identified 3 groups of normal-use, over-use and under-use and examined known factors as putative differentiators between the over-users and the other groups.

**Results:**

After modeling computer screen time according to age, factors linked to over-use were: decreased socialization (OR 3.24, Confidence interval [CI] 1.23–8.55, p = 0.018), difficulty to disengage from the computer (OR 1.56, CI 1.07–2.28, p = 0.022) and age, though borderline-significant (OR 1.1 each year, CI 0.99–1.22, p = 0.058). While psychopathology was not linked to over-use, post-hoc analysis revealed that the link between increased computer screen time and psychopathology was age-dependent and solidified as age progressed (p = 0.007). Unlike computer usage, the use of small-screens and smartphones was not associated with psychopathology.

**Conclusions:**

The results suggest that computer screen time follows an age-based course. We conclude that differentiating normal from over-use as well as defining over-use as a possible marker for psychiatric difficulties must be performed within the context of age. If verified by additional studies, future research should integrate those views in order to better understand the intricacies of computer over-use.

## Introduction

Computer use has become exceedingly prominent in children's and adolescents' lives [1–3], prompting extensive research efforts focusing on the issue of computer usage and computer screen time (CST) [4].

### Psychopathology and Excessive CST

High levels of computer screen usage have been repeatedly linked to violence [2], attention and psychosocial problems [4, 5], reduced parental and peer attachment [6], obesity [7] and poor academic achievements [8]. However, the exact nature of these correlations has yet to be definitively clarified and the causality to be proven. In fact, several studies suggested a reversed or mixed causality for these associations (i.e. psychological difficulties lead to increased CST) [9–12], and others have even hinted at possible beneficial effects [13–15].

Surprisingly, many of the studies lack the context of age when addressing these links, even though all psychological development is age-dependent, and several behaviors surrounding aspects of video-games and other types of screen-usage are no exception [16].

Thus, the ambiguity increases as findings linking increased CST to psychopathology cannot simply be generalized from one age group to other age groups.

### How Much is "OK"?

Whether increased CST leads to psychopathology or just serves as a possible marker for its existence, one should wonder how much CST would either pose a danger to a child's development or signal the need for psychiatric investigation.

Despite the absence of concrete data about what is "expected" or "normal" CST, warnings have been published in both professional and popular literature [17–20]. It seems that there is very limited information about what should be considered as "normal usage". Previous studies have reported mean daily computer usage of 1–3 hours per day between the ages of 8–18 [2, 11], and even up to 5 hours per day [10]. Due to the fast pace of interactive media penetration and evolution, published data may often be out-of-date.

Previous studies delineating CST in children and adolescents, like studies exploring the link between CST and psychopathology, have not truly taken age into account. A study published in 2004 has reported 0.72 hours a day as a mean CST between the ages of 6–11 [3]. However, this finding is not enough to clarify what should constitute a warning sign. Considering that based solely on gender and parental control, it is only to be expected that an 11 years old boy spends more time at his computer than a 6 years old girl [21].

Age-specific data is scarce, usually focuses on narrow age groups [22, 23] and the few studies that address this issue do not provide a range for "normal use", but rather focus on mean CST which comprises both normal and pathological or over-users [21]. In most studies, reliance on children's self-report alone or parents alone (rather than both children and parents) may further complicate attempts to achieve coherent data [1], as children at different ages can potentially bias the report differentially.

CST is not a criterion in the definition of Internet-Gaming-Disorder in the DSM5 [24], which focuses on the pattern of use more than the time spent in front of a screen. However, CST is a common attribute of this disorder [21, 24], probably the most "visible" sign of pathological-use and an easy screening question.

### CST as an Age-dependent Behavior (How Much is Age-Appropriate?)

Many attributes of children are assessed as "normal" or "pathological" based on the context of age, and developmental charts are the prime example for that. Medical students are taught that the correct way to respond to any question in pediatrics is by first inquiring about the age of the child. Thus, the aims of this study were: (1) to examine whether using a model that takes age into account provides a better discrimination between normal and excessive CST and enables identification of a reference "normal range"; (2) to explore whether known behavioral variables that have been previously linked to over–use are still relevant when an age-based approach is used; (3) to study the link between psychopathology and increased CST in the context of age.

## Materials and Methods

### Ethics Statement

The study was approved by the institutional review boards of both centers. Oral consent was given by all parents and children above the age of 13 and was documented by the investigators, as approved by both institutional review boards due to the absence of risk to the participants and to ease the recruitment procedure.

### Participants

The sample was recruited through a large academic children and adolescents outpatient clinic within a central psychiatric center and a specialized developmental pediatrics clinic and was supplemented by word-of-mouth recruitment. Since we hypothesized that age and psychopathology are influential factors, we chose study sites that can provide variability in these parameters: both of these clinics care for a variety of psychopathologies at a wide range of ages. In this manner, we have enriched our study population in order to sample a large proportion of children suffering from psychological or psychiatric difficulties.

Our study consisted of two populations surveyed in two different ways: Data concerning children under the age of 8 was gathered through questionnaires filled by their parents; Data concerning children ages 8–18 years was gathered by pairs of questionnaires; one filled by the child and one by a parent.

82 parents of children 3–8 years old were recruited as well as 103 pairs of parents and children aged 8–18 years.

Inclusion criteria were: (1) age 3–18 years old; (2) Hebrew fluency of the parent and the child; and (3) the presence of at least one computer at home. Exclusion criteria were: (1) diagnosis of a psychotic or autistic spectrum disorder; and (2) child’s major sensory deprivation (e.g. deafness).

### Measures

Information was gathered using questionnaires that were specifically designed for the current study.

The questionnaires were comprised of 3 parts: (1) Demographic and socio-economic information, including functional and academic variables, as well as leisure activities (other than computer use); (2) Information regarding computer use habits, usage during weekdays and weekends, type of usage (e.g. games, social media), parental stands regarding computer use, on-line socialization, non-computer electronic media use (tablet computers, smart phones, game consoles) and difficulty regulating computer use; and (3) Psychiatric screening, using the well-validated Strength and Difficulties Questionnaire (SDQ), a 25-items questionnaire, each rated in a three-point Likert scale ("Not True", "Somewhat True", "Certainly True") for the purpose of psychiatric screening of children and adolescents, which yields specific signals in five different diagnostic spectrums: emotional, hyperactivity, conduct, peer and prosocial difficulties, as well as a general signal of psychopathology [25–27]. Multiple studies examining different populations including international large scale surveys validated the tool as a bone-fide dimensional measure of children's psychological health.

Questions gathering information regarding computer use habits were based on previous studies in this matter [16]. They were constructed by agreement between the researchers and tested for clarity on 4 adults and 6 children.

The two parent's questionnaire versions (3–8 and 8–18 years old) and the child version (8–18 years old) were mostly similar, questions that are age-dependent (e.g. school settings, extra-curricular activities, socialization) were tweaked for better age-matching. In addition, minute changes in the SDQ were implemented according to age groups, as required (http://www.sdqinfo.org) [28]. The full text of a sample questionnaire (in the original Hebrew) is available as supplementary material (S1 Questionnaire).

### Statistical Analysis

In the current study we have tried to shed light on the role of age in the evaluation of CST. Thus, we applied a linear regression model to describe CST as a function of age, in a similar manner to developing a developmental chart.

#### CST as a Factor of Age

A regression model with 95% confidence interval (CI) was applied to the normal ("clean-SDQ") participants (i.e., group without psychopathology). The CI upper and lower limits were defined as the thresholds for over-use and under-use, respectively, thus allowing 3 groups to be formed–normal-use (CST within the 95% CI), over-use (CST above upper 95% CI limit) and under-use (CST below lower 95% CI limit).

Children whose SDQ scores suggested psychopathology, were excluded from the population used to develop the model since many studies linked psychological and psychiatric difficulties with increased CST. Including those subjects in the "age-based CST chart" had the potential to shift the normative curve upwards, thus artificially increasing the upper limits of normal CST.

#### Characterizing the Normal and Psychopathology Groups

The groups were compared using 2-tail T-test for continuous variables and Chi-square test for categorical and ordinal variables, in order to identify possible confounders.

#### Factors Affecting CST

After the psychopathology group was re-introduced, a logistic regression model was applied in order to identify factors that differed between normal and under-users (all subjects with CST below the 95% CI upper limit) and over- users (whose CST exceeds the 95% upper limit), such that only factors contributing to one becoming an over-user would surface, and not all factors related to CST increase. The amount of time spent in different types of usage, reported in a Likert-type scale and grouped into 2 categories (low vs. high use) was compared between the normal and over-users using the Fisher's exact-test for 2 groups. For further in-depth view on the link between age, psychopathology and increased CST, a 2-way-ANOVA model was used.

## Results

### General

90% (167/185) of the questionnaires contained sufficient information to be included (full parental SDQ, elaboration of computer use habits and demographics). 35% of subjects were female, 65% were below the age of 12 years.

### Characteristics of the Normal and Psychopathology Groups

90 participants (54%) had a "Clean-SDQ" (none of the pathological scales were above the cutoff points); and those comprised the normal group. 77 children (46%) had one or more pathological scores of the SDQ scales, comprising the psychopathology group.

Demographics, as well as measures describing non-computer-related behaviors and computer-related behaviors are shown in Table 1.

**Table 1:** Characteristics of the normal and psychopathology groups.

Parameter		Normal group Mean (SD)	Psychopathology group Mean (SD)	p Value
**Demographics**				
	Child age (years)	8.9 (4.5)	9.8 (4.3)	n.s
	Child gender (% males)	50%	78%	<0.001
	Parent gender (% males)	27%	25%	n.s
	Parental marital status (% married)	95%	76%	0.005
	Number of electronic media devices (television, computers, consoles, tablets)	5.9 (2.8)	5.8 (2.5)	n.s
	Socio-economic status (people per room)	1.2 (0.4)	1.5 (1.4)	n.s
	Parents' education (high-school, academic)	17%, 83%	20%, 80%	n.s
**Non-Computer-related habits**				
	Extra-Curricular Activities (% Participation)	84%	61%	0.004
	Social Involvement (% frequent participation)	74%	63%	n.s
	Average school grades	91.4	89.4	n.s
	Behavioral school grade ^a^	95.9	92.9	0.099
	Daily number of TV hours	1.7 (1.2)	2.2 (1.3)	0.017
	Daily hours of smartphone use (including talking, playing, texting)	1.6 (2.1)	1.3 (1.9)	n.s
**Computer-Related Habits**				
	Number of days per week using a computer	4.2 (2.9)	5.1 (2.6)	0.038
	Average daily computer screen time (hours)	1.5 (2.0)	2.6 (2.7)	0.004
	Average daily small-screen gaming (hours)	0.61 (0.93)	0.64 (0.85)	n.s
	Number of Facebook friends	195.7 (314.9)	117.7 (197.4)	n.s
	Difficulty in stopping computer-use (% with difficult disengagement)	38%	59%	<0.001
	Parents' approach to legitimate computer uses (prevent, control, allow) ^b^	28%, 62%, 10%	21%, 72%, 7%	n.s
	Parents' approach to legitimate CST (no rules, general rules, defined rules)	19%, 70%, 11%	14%, 73%, 13%	n.s

Group comparisons were performed using T-test for continuous measures, and χ^2^ for categorical measures.

^a^ A grade given according to active participation in class, school attendance etc.

^b^ Parents were asked to choose the best description to their approach towards legitimate computer use, ranging from "discouraging any kind of use (except school assignments)", "have no objections to different uses (including social media or games) as long as reasonably balanced" and "have no objection to different uses, and do not regulate the child's way of using the computer".

In general, most children and adolescents were found to use computers 1–3 hours a day. A significant and substantial difference in CST was found between males and females (2.4 vs. 1.5 hours per day, respectively, p = 0.011). Those who had an indication of behavioral or emotional difficulties used computers for a longer time (mean daily 2.6 hours compared to 1.5 hours; p = 0.004), and found it more difficult to disengage from the computer (59% compared to 38%; p<0.001). There were no significant differences in parental attitudes between the two groups. A similar pattern was observed for watching television (TV): most children and adolescents watch TV for 1–3 hours a day, but those children and adolescents with difficulties watch more (2.2 vs. 1.7 hours per day respectively, p = 0.017). Children without psychopathology were significantly more involved in extra-curricular activity (84% as compared to 61%; p = 0.004). Demographically, the psychopathology group was comprised of a larger proportion of males and had fewer married parents. There were no significant differences in socio-economic status between the groups.

In order to estimate CST, parental reports were used to enable integration of data concerning children throughout the age range of the study. A significant positive correlation between parental and adolescent CST reporting (r = 0.8, p<0.01) and a non-significant difference demonstrated that these two measures are sufficiently similar (Parents mean daily CST 3.5 hours, ±2.6; Children mean daily CST 3.5 hours, ±2.8; p>0.1).

**Fig 1:**
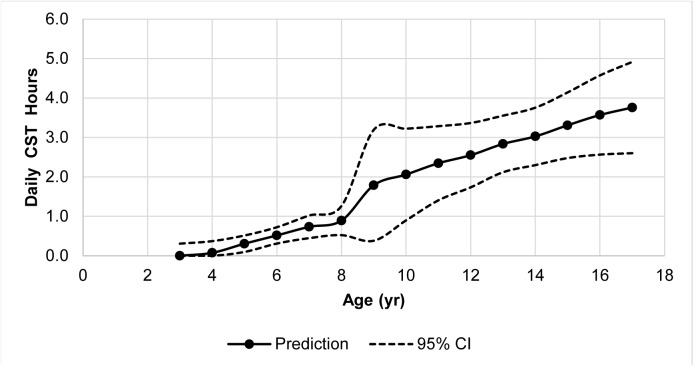
Predicted average daily computer screen time hours based on the normal group age regression model. CST–Computer screen time.

An age-based regression model of parental-reported CST was applied separately to the younger and to the older subgroups of the normal group (the group without psychopathology, based on SDQ scores). According to the model, lower and upper thresholds were determined, using 95% CI specific for each age (Fig 1).

Following generation of the regression model and establishment of the limits of normal-usage, the SDQ-positive participants (psychopathology group) were added. The CST of the entire study-population (including the psychopathology group) was compared to the thresholds, thus creating the 3 groups of normal-use, under-use and over-use. When examining the correlation between CST and age as a continuous regression model, a one year increase of age increased CST for young children (3–8 years old) by 12.2 minutes a day (CI 5.4–19.1 minutes) and for older children and adolescents (8–18) by 21 minutes (CI 10.2–31.8 minutes).

### Factors affecting Computer Over-Use

Table 2 presents univariate analysis of the variables entered into the logistic regression, compared between the over-use group and the combined normal-use and under-use groups.

**Table 2:** Characteristics of the over-use versus normal & under-use groups, univariate analysis. Group comparisons were performed using T-test for continuous measures, and χ^2^ for categorical measures.

Parameter		Over-use group Mean (SD)	Normal & under-use group Mean (SD)	p Value
**Demographics**				
	Child age (years)^a^	10.8 (4.5)	8.9 (4.4)	0.017
	Child gender (% males)	74.4%	57.7%	0.052
	Number of electronic media devices (television, computers, consoles, tablets)	6.2 (2.7)	5.8 (2.7)	n.s
	Socio-economic status (people per room)	1.2 (0.3)	1.4 (1.1)	n.s
	Parents' education (high-school, academic)	23%, 77%	17%, 83%	n.s
**Non-Computer-related habits**				
	Extra-Curricular Activities (% Participation)	71.2%	66.7%	n.s
	Social Involvement (% frequent participation)	79.5%	52.4%	0.001
	Average school grades	86.2 (13.3)	92.1 (7.3)	0.041
	Behavioral school grade ^b^	93.6 (8.6)	94.9 (8.1)	n.s
	Daily number of TV hours	2.6 (1.5)	1.8 (1.1)	<0.001
	Daily hours of smartphone use (including talking, playing, texting)	1.7 (2.2)	1.4 (1.9)	n.s
**Computer-Related Habits**				
	Average daily small-screen gaming (hours)	0.9 (1.3)	0.56 (0.8)	n.s
	Number of Facebook friends	208.2 (309.5)	157.0 (267.6)	n.s
	Difficulty in stopping computer-use (% with difficult disengagement)	67.4%	41.5%	0.004
	Parents' approach to legitimate computer uses (prevent, control, allow) ^c^	6%, 66%, 28%	15%, 68%, 18%	n.s
	Parents' approach to legitimate CST (no rules, general rules, defined rules)	19%, 74%, 7%	15%, 70%, 15%	0.053
**Psychiatric Screening**				
	Positive SDQ Signal	59.5%	41.3%	0.041

CST–Computer Screen Time; SDQ–Strengths and Difficulties Questionnaire.

^a^ Over-use was defined as excessive use (>95% CI) per specific age.

^b^ A grade given according to active participation in class, school attendance etc.

^c^ Parents were asked to choose the best description to their approach towards legitimate computer use, ranging from "discouraging any kind of use (except school assignments)", "have no objections to different uses (including social media or games) as long as reasonably balanced" and "have no objection to different uses, and do not regulate the child's way of using the computer".

To evaluate possible interactions between variables, we examined the correlations of all continuous factors prior to their introduction into the logistic regression. These are presented in Table 3.

**Table 3:** Correlations between variables entering the logistic regression model.

Pearson correlation (p Value)	Age	Socio-economic	Number of devices	School Grades	School behavior	Smartphone use	Television hours	Small screens use	Computer Screen Time	Facebook Friends
Age	-									
Socio-economic	0.03 (n.s)	-								
Number of devices	0.41** (<0.001)	-0.13 (n.s)	-							
School Grades	-0.39* (<0.001)	0.18 (0.09)	-0.11 (n.s)	-						
School behavior	-0.07 (n.s)	0.02 (n.s)	-0.13 (n.s)	0.45** (<0.001)	-					
Cellphone use	0.3* (0.007)	-0.11 (n.s)	0.23* (0.045)	-0.27* (0.021)	0 (n.s)	-				
Television Hours	0.35* (<0.001)	-0.06 (n.s)	0.28* (<0.001)	-0.26* (0.015)	-0.08 (n.s)	0.17 (n.s)	-			
Small screens use	0.39* (<0.001)	-0.05 (n.s)	0.34* (<0.001)	-0.15 (n.s)	-0.19 (0.091)	0.43** (<0.001)	0.18* (0.026)	-		
Computer screen time	0.68*** (<0.001)	0.04 (n.s)	0.25* (0.001)	-0.42** (<0.001)	-0.15 (n.s)	0.20 (0.073)	0.39* (<0.001)	0.31* (<0.001)	-	
Facebook Friends	0.40** (<0.001)	-0.09 (n.s)	0.3* (0.017)	-0.27* (0.038)	0.09 (n.s)	0.57** (<0.001)	0.19 (n.s)	0.23 (0.078)	0.24 (0.053)	-

* Significant correlation <0.4.

** Significant correlation 0.4–0.6.

*** Significant correlation >0.6.

Logistic regression with backwards elimination was applied to identify the characteristics of over-users as compared to normal and under-users. Two factors surfaced as significant characteristics of over-users: difficulty to disengage computer use, which is a feature of compulsivity (OR 1.56, CI 1.07–2.28, p = 0.022) and decreased socialization with friends (OR 3.24, CI 1.23–8.55, p = 0.018). In addition, a borderline-significant factor of age (OR 1.1 for each progressing year, CI 0.99–1.22, p = 0.058) also surfaced. The model, based on these 3 variables, yielded r^2^ = 0.204.

Factors entered into the model that have not yielded significance were gender, socio-economic status, number of electronic media devices, parental education, school grades, extra-curricular activities, amount of TV use, weekdays vs. weekends patterns of use, academic performance, smartphone use (including talking, texting and gaming), small-screen gaming (smartphones and tablets), and parental stand regarding computer use.

No significant difference was found on any of the types of usage inquired (games, social media, small screens and content) between the groups.

Several variables that were significant in univariate analysis, when comparing the over-use group with the normal-use group, were no longer significant in the logistic regression: school grades, TV hours and positive SDQ signal. Gender and parents' approach towards CST were borderline significant in the univariate analysis and were also no longer significant in the backward step regression.

**Fig 2:**
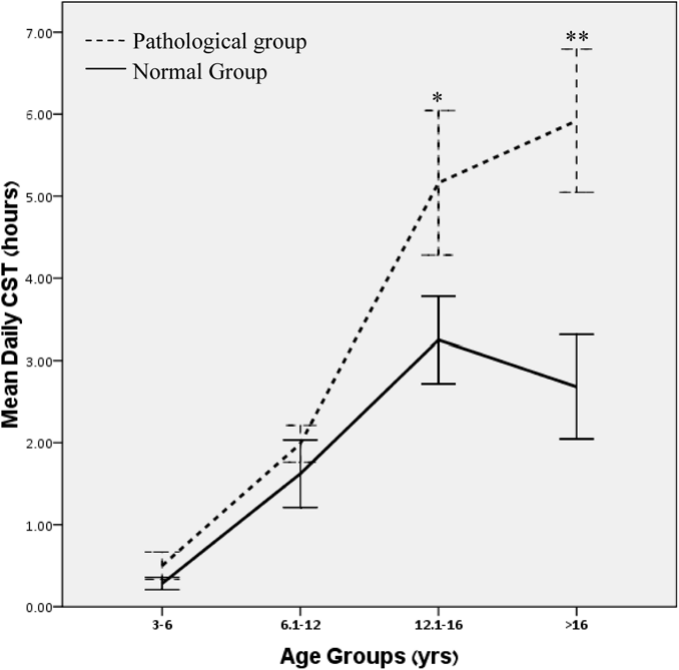
Mean daily computer screen time (hours) of the normal and psychopathology groups, at different developmental stages. * Borderline significant difference; ** Significant difference; Error bars represent ±1 standard error. CST–Computer Screen Time.

This analysis showed that SDQ signals were not significant characteristics of over-users. As this finding is incongruent with the literature on this subject, as well as with our own findings which have shown a major and significant difference in CST (Table 1) between the normal and psychopathology groups, we analyzed the data further, using our developmental approach. We divided the participants into 4 age-groups, according to common developmental stages: preschoolers (3–6y), latency (6–12y), adolescence (12–16y) and late adolescence (16–18y). The differences in CST between the normal and the psychopathology groups grew in scope (Fig 2) and acquired statistical significance with age (preschoolers 0.3 vs. 0.5, p>0.1; latency 1.6 vs. 2.0, p>0.1; adolescence 3.2 vs. 5.2. p = 0.076; late adolescence 2.7 vs. 5.9 hours, p = 0.012).

Further analysis, using 2-way ANOVA, revealed significant effects for the age groups (p<0.001), psychopathology (p<0.001) and the interaction between age and psychopathology (p = 0.007).

## Discussion

In western society most children start playing computer games in early childhood [3]. In accordance with existing literature, our study found a correlation between having emotional or behavioral difficulties and spending more time using computers [2, 4–6] and finding it harder to disengage from the computer [29]. Interestingly, this difference is evident only when assessing computer screen time: no difference was found in the use of smartphone (for all possible uses–talking, texting and gaming) or small-screen gaming (smartphones and tablets). This finding might suggest that the use of computers per-se has special attributes that lead to the different usage between the normal and psychopathological group. A possible explanation might be the common and casual use of small-screen devices, rather the focused activity on computers.

In the current study we have shown that the definitions for CST norms should be evaluated in the context of age. Our results suggest that the definition of computer over-use is meaningful only when put in the perspective of age. A practical example of this insight would be a child who plays 2.5 hours a day; this information by itself is of very limited significance. Our data suggests that within certain age ranges (15–18 years) this amount of usage is within the range of time spent by normative teens and according to our findings, is not linked to social or academic dysfunction. On the other hand, the same amount of usage might serve as a warning sign in an 8 year old.

Though our sample is not sufficient for defining rigorous thresholds, we have tried to establish the feasibility of creating a "developmental CST chart". If verified in larger-scale studies, it is a step towards creating standardized guidelines that may address the unmet need for guidance for both families and clinicians. Such charts can assist in identifying instances in which usage is "out of range" and possibly deserves more evaluation.

Our data offers an insight into the nature of the previously described link between psychopathology and increased CST [4]. We found that this link is age-based: the association between emotional and behavioral difficulties and increased CST is created and solidifies as age progresses. Along with the importance of age, not only as a baseline reference factor but also as a contributor to the risk of being an over-user, it seems that late adolescence is the main period for such over-use, especially when psychopathology is present.

Gender should be taken into account: in previous studies [1, 11], as in our own, boys’ average CST was found to be higher than girls. It should be noted that while not significant in the regression model, direct comparison between the over-use and the normal-use groups found it to be borderline significant (p = 0.052). A possible explanation would be that while gender is strongly associated with CST, it is not a significant factor associated with being an over-user.

SDQ measures did not reach statistical significance in the regression model despite the substantial significant differences in CST found between the normal (clean-SDQ) and the SDQ-positive groups. However, other measures that are strongly linked to psychopathology were significant: lower sociability and difficulty to regulate computer use were associated with increased risk for over-use. It is possible that these two features represent broader phenotypes present in children that do not cross the SDQ cutoff. It should also be mentioned that both these measures have been reported in previous studies as linked to CST [1, 3, 11]. It seems that those factors, as well as age, overshadow the role of psychiatric pathology in computer over-use.

Due to our limited sample size we could not examine each SDQ scale individually, thus the psychopathology group is heterogeneous (i.e. externalization vs. internalization) and comprises difficulties that potentially affect differentially and conceal the link to psychopathology.

However, the three factors that were in significant correlation with CST (age, sociability and regulation) account for only 20.4% of the variance between the normal-use and over-use in the sample, suggesting that other factors, either not examined in this study or failing to reach statistical significance, play an additional considerable role.

Our study was not designed to address the important questions about the causal relationship between psychological difficulties and CST. However, as many of the difficulties arise in early childhood (examples include both categorical diagnosis such as ADHD and dimensional difficulties such as social difficulties), there is a lag-time between the development of difficulties and that of computer over-use. It seems that as age progresses, psychopathologies are more likely to manifest through increased CST. This finding can contribute to the understanding of the nature of the link between psychiatric difficulties and computer use.

The existence of the under-use group deserves attention, though our study was not designed to investigate this group. Recently it has been suggested that no-use, as well as over-use, may be linked to emotional or behavioral adverse effects [5].

An interesting incongruence with prior studies was lack of association with socio-economic measures [30], including parental education, home crowdedness (people per room) and even computer and console abundance. This may be explained by the meteoric rise in the abundance of reasonably priced entertainment-technology and the general acceptance of its use in society. It may also suggest the need for repeatedly evaluating norms when studying a rapidly changing "socio-cultural" behavior.

The lack of association between parents' self-declared stands regarding type of use and time limits to a child's chances of being an "over-user" bears possible importance if verified in future larger scale studies. Some consider increased CST as an educational failure, related to over liberal parental views or neglect. It seems that age and psychiatric difficulties are better predictors of CST than parental attitudes or socio-economic capabilities. This raises a possible need to re-evaluate some of the current guidelines for parents' counselors [18, 31, 32].

Future studies are needed in order to verify this observation, emphasizing different developmental stages.

We should note that aside from a positive SDQ signal, which was previously discussed, two additional variables were significant on the univariate analysis between the over-users and normal-users, but did not surface on the regression model. These included television hours and school grades. Both variables are correlated with the continuum of CST, yet according to the regression model were not associated with the extreme of being an over-user. Both of the variables are correlated with age, which, according to the regression, seems to be a more prominent factor.

### Limitations and future directions

As previously mentioned, this was a pilot study with a limited sample size. A larger sample would enable the verification of our findings and enhance the resolution of both CST norms as well as factors related to it (such as the role of each SDQ scale). A second limitation is the possibility of selection-bias, as the population was not an organized cohort (such as a school or residential area), so it is possible that the parents and children who were willing to participate represent a subset with unique features, such as increased CST. The accordance of the basic data gathered (e.g. screen time, clinical correlations) with findings in previous studies on this topic supports the validity and generalizability of our findings. A third limitation would be the reliance on SDQ as the sole classification tool. While not reliable as a full psychiatric evaluation, the SDQ was substantiated as a reliable screening tool [26, 27, 33] and has been used repeatedly for the purpose of classification of psychopathology [34–36]. A final limitation is, similarly to many articles published on this matter, the reliance on reported data and not direct observation, thus allowing recall bias to affect the results. We attempted to overcome this obstacle by relying on both self-reports and parental reports. However future studies might benefit from relying on usage monitoring, especially when measuring smartphones and small-screen devices as their use is more intermittent (usually comprising of dozens of few-minutes use over a day), casual and non-focused, making self-report quantification subject to substantial biases.

Computer usage has become an integral part of the current way of life, and probably should be considered an integral part of the social fabric of everyday life, especially in children and adolescents. Furthermore–children are encouraged to use computers for certain purposes (e.g. use of electronic databases and forums for school assignments), and several studies attribute cognitive and psychosocial advantages to computer games [5, 13–15]. As it is not realistic to approach computer-use with utter de-legitimization and prohibition, it is important to understand what is normative and what should be considered as an out-of-norm warning sign, requiring further evaluation.

Thus, "screen-time" should be used as a screening question, a doorway to a much more detailed inquiry about the content of this usage, as well as digital behavior as a whole [37].

## Conclusion

Assessing computer usage in the context of age can be beneficial to both clinicians and parents, and may help clarify some of the discrepancies in current literature. Being an over-user is linked to paucity of social interactions and difficulty to regulate computer use. It seems that emotional and behavioral difficulties are linked as well, but only in later adolescence.

Further studies are needed to reaffirm these findings and hopefully enhance the detailed understanding of over-use characteristics, allowing guidelines and definitions to outline the boundaries of normal and adaptive use.

## Supporting Information

S1 Questionnaire Questionnaire for parents, ages 8–18, Hebrew. (DOC)
