# Supplementary material for: Evaluating Computer Screen Time and Its Possible Link to Psychopathology in the Context of Age: A Cross-Sectional Study of Parents and Children
Source: PLoS One. 2015 Nov 4;10(11):e0140542. doi: 10.1371/journal.pone.0140542 (PMC4633223; doi:10.1371/journal.pone.0140542)
Supplement: S1 Questionnaire — (DOC) [file pone.0140542.s001.doc]

קוד: _____ - _____-818P- ג-1.00/ת-14.04

**משחקי מחשב – לא רק משחק...**

**גרסת ההורים**

**שלום רב!**

לפניכם שאלון הניתן לכם במסגרת מחקר הנערך על שימוש במשחקי מחשב בילדים ונוער (גילאי 8-18).

השאלון מורכב מחמישה חלקים, כאשר כל חלק עוסק בנושא אחר:

- החלק הראשון עוסק בשאלות כלליות (כגון מין, גיל וכו')
- החלק השני עוסק בפעילות הלימודים והפנאי של ילדך
- החלק השלישי עוסק בהרגלי השימוש של ילדך במחשב
- החלק הרביעי עוסק במשחקי המחשב האהובים על ילדך
- החלק החמישי עוסק מעט באופי של ילדך

השאלון מעט ארוך, אבל חשוב מאוד לנסות לענות על כל השאלות המופיעות בו.

כמו כן, חשוב מאוד לנסות ולענות בכנות רבה ככל שניתן על השאלות.

בנוסף, נבקש שתמלא את השאלון ללא סיוע של ילדך, ובמקרה וילדך זקוק לסיוע במילוי השאלון שלו, נודה לך אם תסיים למלא את השאלון שלך קודם לכן.

המידע הוא אנונימי, והמידע הנאסף בו נשמר בסוד ואינו חשוף לאיש פרט לחוקרים.

השאלון מנוסחות כולן בלשון זכר, אך הן מיועדות לגברים ולנשים כאחד. ההתייחסות לילדים גם היא בלשון זכר, וגם כאן כמובן מיועדות השאלות לבניכם ובנותיכם כאחד.

אנחנו מאוד מודים לך ומעריכים את ההשתתפות ואת מילוי השאלון.

**תודה רבה!**

**חלק ראשון**

**בחלק זה תשאל שאלות כלליות לגביך, כדי שנכיר מעט את הרקע שלך.**

1. מין
   1. זכר
   2. נקבה
2. תאריך לידה _______
3. מצב משפחתי
   1. נשוי
   2. גרוש
   3. אלמן
   4. רווק
4. מספר הילדים _______
5. כמה אנשים גרים בבית _______
6. כמה טלויזיות יש בבית ______
7. כמה מחשבים יש בבית ______
8. כמה קונסולות יש בבית (PS, Wii, Xbox) ______
9. מספר חדרי מגורים בבית (כולל חדרי שינה, חדר עבודה וכיו"ב, לא כולל סלון) ______
10. השכלה
    1. עממית
    2. תיכונית
    3. תיכונית ותעודת בגרות
    4. על-תיכונית מקצועית
    5. אקדמאי – תואר ראשון
    6. אקדמאי – תואר שני או שלישי

**חלק שני**

**בחלק זה נשאל תשאל שאלות על סדר היום של ילדך.**

1. האם הוא חבר בתנועת נוער, חוגים או ארגון התנדבותי?
   1. כן, פירוט _____________________________________
   2. לא
2. האם הוא עוסק בספורט במשך השבוע?
   1. כן, באופן עצמאי (כגון חדר כושר או משחקי כדורסל)
   2. כן, באופן מקצועי (כגון קבוצות מקצועיות)
   3. לא
3. מספר השעות (בממוצע) בו הוא צופה בטלוויזיה ביום הוא __________
4. יש לו טלפון סלולארי מגיל _______ (אם אין לו טלפון סלולארי משלו, אנא כתוב 0)
5. מספר השעות (בממוצע) בו הוא משתמש בטלפון סלולארי (כולל לדבר, לשחק, להוריד דברים, לשלוח סמסים) ביום הוא _______ (אם אין ברשותו טלפון סלולארי, ציין 0)
6. הציון שלו בחשבון/מתמטיקה בתעודה האחרונה היה ________
7. הציון שלו באנגלית בתעודה האחרונה היה ________
8. הציון שלו בתנ"ך בתעודה האחרונה היה ________
9. הציון שלו בהתנהגות בתעודה האחרונה היה ________
10. הוא נפגש עם חברים:
    1. רק בביה"ס או באירועים מיוחדים
    2. בביה"ס ובסופי-שבוע
    3. בביה"ס, סופי שבוע, ולעיתים קרובות גם אחרי הלימודים

**חלק שלישי**

**בחלק זה תשאל על הרגלי השימוש במחשב של ילדך.**

1. הגיל בו התחיל ילדך להשתמש במחשב הוא _________
2. מספר הימים בשבוע בו ילדך משתמש במחשב הוא _______
3. מספר השעות בו הוא נמצא מול המחשב או הקונסולה ביום רגיל (ממוצע) הוא ______
4. מספר השעות בו הוא נמצא מול המחשב או הקונסולה ביום חופש הוא _______
5. מספר החברים שלו שהכיר באינטרנט אבל לא פגש מעולם ________ (אם אין כאלה, אנא ציין 0)
6. מספר החברים שלו שהכיר באינטרנט אבל היום הם חברים שלו גם במציאות ________ (אם אין, ציין 0)
7. מספר החברים שלו בפייסבוק ______ (אם אין פייסבוק, אנא ציין 0)
8. הוא משחק בקונסולת מחשב:
   1. לא
   2. כן – Wii (וויי)
   3. כן – PlayStation (פלייסטיישן) או Xbox (אקסבוקס)
   4. כן – PSP/GameBoy (גיים-בוי)
   5. כן – iPad (אייפד) או מחשב Tablet אחר
9. מספר השעות בו משחק במשחקים בטלפון חכם או מחשב Tablet (iPhone, iPad) ביום ממוצע הוא _____
10. האם קשה לו לפעמים להפסיק את השימוש במחשב כאשר הוא צריך ללכת לישון, לאכול או להכין שיעורים?
    1. כמעט תמיד
    2. לעיתים קרובות
    3. לפעמים
    4. לעיתים רחוקות
    5. אף פעם

עבור כל פריט, סמן כמה זמן לדעתך מקדיש לו ילדך זמן (מתוך זמן השימוש הכולל שלו במחשב):

|  |  | **בכלל לא** | **מעט זמן** | **די הרבה זמן** | **רוב הזמן** |
| --- | --- | --- | --- | --- | --- |
| 11. | משחקי מחשב או משחקים בקונסולה (כגון Xbox) | □ | □ | □ | □ |
| 12. | גלישה באינטרנט, YouTube (יוטיוב) | □ | □ | □ | □ |
| 13. | הורדת סרטים או מוזיקה | □ | □ | □ | □ |
| 21. | משחק בטלפון חכם או מחשב Tablet (iPhone, iPad) | □ | □ | □ | □ |
| 14. | קשר עם חברים - פייסבוק, אי-מייל, מסנג'ר, סקייפ | □ | □ | □ | □ |
| 15. | כתיבת עבודות ושיעורי בית לביה"ס | □ | □ | □ | □ |
| 16. | השתתפות בפורומים וקבוצות דיון | □ | □ | □ | □ |
| 17. | תיקון תקלות ושדרוג המחשב | □ | □ | □ | □ |
| 18. | יצירה ותחזוקה של תכנים שלו (בלוג, אתר) | □ | □ | □ | □ |

1. מה עמדתך לגבי אופן השימוש הרצוי של ילדך במחשב?
   1. אני משתדל למנוע או לצמצם עד כמה שאפשר את השימוש במחשב
   2. אני תומך בשימוש במחשב לצרכי לימוד, אבל מתנגד לשימוש לצורך חברה, פנאי או משחקים.
   3. אני תומך בשימוש במחשב לצרכי לימוד וכן לתקשורת עם חברים או פנאי (YouTube, פייסבוק, הורדות מוזיקה) במידה והדבר נעשה באופן סביר, אך מתנגד לשימוש במחשב לצורך משחקים.
   4. אין לי התנגדות לכלל שימושי המחשב (לימוד, פנאי, חברים, משחקים), כל עוד נעשה באופן סביר.
   5. אין לי התנגדות לשימוש במחשב על כל שימושיו (לימוד, פנאי, חברים, משחקים), ואיני מבקר את אופן השימוש של הילד.
2. מהם הכללים בבית לגבי זמני השימוש במחשב?
   1. אין הגבלה על זמני השימוש שהילד עושה במחשב.
   2. אין כלל ברור, אבל כאשר אני חש שהשימוש במחשב מוגזם, אני מעיר ומתייחס / מונע את המשך השימוש (מחק את המיותר), והדבר קורה כמה פעמים ביום / פעם ביום / כמה פעמים בשבוע / פעם בשבוע או פחות (מחק את המיותר).
   3. ישנם כללים ברורים לגבי זמני השימוש: בימי בית ספר ______ שעות, בסופ"ש _____ שעות, בחופשים _____ שעות, וכאשר יש חריגה מהכלל אני בד"כ מתעלם / מעיר ומתייחס / מונע את המשך השימוש (מחק את המיותר), והדבר קורה כמה פעמים ביום / פעם ביום / כמה פעמים בשבוע / פעם בשבוע או פחות (מחק את המיותר).

**חלק רביעי**

**בחלק זה נשאל אותך על משחקי המחשב בהם ילדך אוהב לשחק.**

עבור כל פריט, סמן עד כמה אתה מסכים עם הכתוב בו, כאשר האפשרויות הן:

"לא מסכים כלל" – כאשר אתה מרגיש כי הכתוב במשפט כמעט אף פעם אינו נכון.

"לא כל-כך מסכים" – כאשר אתה מרגיש כי הכתוב במשפט בדרך כלל אינו נכון.

"קצת מסכים" – כאשר אתה מרגיש כי הכתוב במשפט לפעמים נכון.

"מסכים" – כאשר אתה מרגיש כי הכתוב במשפט בדרך כלל נכון ומתאים.

"מסכים מאוד" – כאשר אתה מרגיש כי הכתוב במשפט מאוד מתאים ונכון כמעט תמיד.

השתדל לענות על כל הפריטים בצורה הטובה ביותר, גם אם אתה לא לגמרי בטוח.

סמן את התשובות בהתאם לאיך שהדברים היו עבור ילדך בשנה האחרונה.

מספרי השאלות אינם עוקבים בשל ההתאמה לשאלון עליו עונים ילדיכם. עמכם הסליחה.

|  |  | **לא מסכים כלל** | **לא כל-כך מסכים** | **קצת מסכים** | **מסכים** | **מסכים מאוד** |
| --- | --- | --- | --- | --- | --- | --- |
| 2. | לעיתים הוא מוצא עצמו משחק שעות במחשב בלי לשים לב לזמן שעבר | □ | □ | □ | □ | □ |
| 4. | הכינוי של הדמות שלו במשחקים שונים הוא אותו שם או שם דומה | □ | □ | □ | □ | □ |
| 5. | לעיתים הוא מוציא כסף או מבקש כסף על מנת לרכוש יכולות או חפצים לדמויות אותן משחק | □ | □ | □ | □ | □ |
| 7. | כשיש לו אפשרות בחירה, הדמות שלו במשחק היא מאותו המין כמוהו | □ | □ | □ | □ | □ |
| 8. | הוא מעדיף לשח ק משחקי מחשב עם שחקנים נוספים | □ | □ | □ | □ | □ |
| 9. | הוא מעדיף לשחק משחקי מחשב עם חברים הנמצאים פיזית לידו | □ | □ | □ | □ | □ |
| 11. | הוא אוהב משחקים הנמשכים זמן רב (שבועות) עד שמסיים אותם | □ | □ | □ | □ | □ |
| 13. | הוא משחק משחקי מחשב בהם אין אפשרות לנצח, אלא מטרת המשחק היא גדילה והתפתחות (כגון Sims או SimCity) | □ | □ | □ | □ | □ |
| 16. | הוא אוהב משחקי בהם הוא מגלם דמות (כגון Worlds of Warcraft) | □ | □ | □ | □ | □ |
| 17. | הוא אוהב משחקי מכות (כגון Tekken) | □ | □ | □ | □ | □ |
| 18. | הוא אוהב משחקי אסטרטגיה (Civilization, Starcraft) | □ | □ | □ | □ | □ |
| 19. | הוא אוהב משחקי ירי (First Person Shooter/Third כגון Call of Duty, Halo, Dead Space) | □ | □ | □ | □ | □ |
| 20. | הוא אוהב משחקי עיצוב ותכנון (כגון SimCity, The Sims, Tycoon) | □ | □ | □ | □ | □ |
| 21. | הוא אוהב משחקי פעולה (Pinball, Bubbles, Fruit Ninja, משחקי פלטפורמות כגון Super Mario) | □ | □ | □ | □ | □ |
| 22. | הוא אוהב משחקי הרפתקאות (כגון GTA, Zelda, Red Dead) | □ | □ | □ | □ | □ |
| 23. | הוא אוהב משחקי סימולציה (מכוניות ומרוצים, מטוסים וכדומה) | □ | □ | □ | □ | □ |
| 24. | הוא אוהב משחקי ספורט (כגון FIFA, NBA) | □ | □ | □ | □ | □ |
|  |  |  |  |  |  |  |
|  |  | **לא מסכים כלל** | **לא כל-כך מסכים** | **קצת מסכים** | **מסכים** | **מסכים מאוד** |
| 25. | הוא אוהב משחקים אקטיביים (כגון Guitar hero, Wii Sports) | □ | □ | □ | □ | □ |
| 26. | הוא מתפאר בהישגי במשחק גם בפני אנשים שלא מכירים את המשחק | □ | □ | □ | □ | □ |
| 27. | נראה שכאשר הוא מצליח במשחק, הוא חש טוב יותר עם עצמו | □ | □ | □ | □ | □ |
| 28. | נראה שכאשר הוא מפסיד במשחק, הדבר משפיע על מצב רוחו גם מחוץ למשחק | □ | □ | □ | □ | □ |

1. הוא מעדיף שתוכן המשחק יהיה קשור ל:
2. פנטזיה – לוחמים, מפלצות וכשפים
3. מדע בדיוני – חייזרים, חלל, טכנולוגיה
4. מלחמה
5. פשע
6. אחר ________
7. לא משנה לו עולם התוכן
8. הוא מעדיף שהאויבים שלו במשחק יהיו ויראו כמו:
9. אנשים
10. אנשים רעים (פושעים, נאצים)
11. לא אנושיים (מפלצות, חייזרים, רובוטים, חיות טרף)
12. לגמרי לא משנה לו
13. בין הדמויות הבאות, הדמות שהוא הכי אוהב ומעדיף להיות היא
14. לוחם
15. קוסם
16. קשת
17. גנב
18. בלש או שוטר
19. פושע
20. לא משנה לו, או שהוא משנה לעיתים די קרובות

אנא סמן מה יחסו של ילדך למשחקים הבאים (ניתן לרשום משחקים נוספים בהם שיחק ואינם מצוינים פה)

|  | **שם המשחק** | **לא מכיר** | **שונא** | **לא כל-כך אוהב** | **קצת אוהב** | **אוהב מאוד** |
| --- | --- | --- | --- | --- | --- | --- |
| 1. | GTA (Grand Theft Auto) | □ | □ | □ | □ | □ |
| 2. | Worlds of Warcraft | □ | □ | □ | □ | □ |
| 3. | Tekken | □ | □ | □ | □ | □ |
| 4. | Call of Duty | □ | □ | □ | □ | □ |
| 5. | Maple Story | □ | □ | □ | □ | □ |
| 7. | Starcraft | □ | □ | □ | □ | □ |
| 8. | FIFA | □ | □ | □ | □ | □ |
| 9. | Halo | □ | □ | □ | □ | □ |
| 10. | Super Mario | □ | □ | □ | □ | □ |
| 11. | Wii Sports | □ | □ | □ | □ | □ |
| 12. | Just Dance | □ | □ | □ | □ | □ |
| 13. | Guitar Hero | □ | □ | □ | □ | □ |
| 14. |  | □ | □ | □ | □ | □ |
| 15. |  | □ | □ | □ | □ | □ |
| 16. |  | □ | □ | □ | □ | □ |

**חלק חמישי (ואחרון...)**

**בחלק זה נשאל אותך קצת על האופי של ילדך, ועל איך הוא מסתדר עם אנשים בכלל וחברים בפרט.**

עבור כל פריט, סמן אם הוא "לא נכון", "נכון במידה מסוימת" או "נכון מאוד" לגביו. השתדל לענות על כל הפריטים בצורה הטובה ביותר, גם אם אתה לא לגמרי בטוח.

סמן את התשובות בהתאם לאיך שהדברים היו עבור ילדך בששת החודשים האחרונים.

|  |  | לא נכון | נכון במידה מסוימת | נכון מאוד |
| --- | --- | --- | --- | --- |
| 1. | הוא מתחשב ברגשות של אנשים אחרים | □ | □ | □ |
| 2. | הוא חסר מנוחה, אינו יכול להישאר רגוע לאורך זמן | □ | □ | □ |
| 3. | יש לו הרבה כאבי ראש, כאבי בטן או מחלות | □ | □ | □ |
| 4. | הוא בדרך כלל מתחלק עם אחרים בדברים, למשל דיסקים, משחקים ואוכל | □ | □ | □ |
| 5. | הוא מתרגז מאוד ולעתים קרובות מאבד שליטה | □ | □ | □ |
| 6. | הוא מעדיף להיות לבד ולשחק לבד | □ | □ | □ |
| 7. | הוא בדרך כלל עושה מה שמהבוגרים מבקשים ממנו | □ | □ | □ |
| 8. | הוא דואג הרבה | □ | □ | □ |
| 9. | הוא מסייע אם מישהו נפגע, מודאג או מרגיש חולה | □ | □ | □ |
| 10. | כמעט תמיד הוא נע בעצבנות או מתפתל | □ | □ | □ |
| 11. | יש לו חבר אחד טוב או יותר | □ | □ | □ |
| 12. | הוא רב הרבה, הוא יכול לגרום לאנשים אחרים לעשות את מה שהוא רוצה | □ | □ | □ |
| 13. | לעיתים קרובות הוא לא מאושר, מדוכא, או מרבה לבכות | □ | □ | □ |
| 14. | אנשים אחרים בגילו בדרך כלל מחבבים אותו | □ | □ | □ |
| 15. | הוא מוסח בקלות, הוא מתקשה להתרכז | □ | □ | □ |
| 16. | הוא "נצמד" למבוגר במצבים חדשים, הוא מאבד ביטחון בקלות | □ | □ | □ |
| 17. | הוא נחמד לילדים קטנים ממנו | □ | □ | □ |
| 18. | הוא משקר או מרמה לעיתים קרובות | □ | □ | □ |
| 19. | ילדים אחרים או אנשים צעירים נטפלים אליו או מאיימים עליו | □ | □ | □ |
| 20. | לעתים קרובות הוא מציע לעזור לאחרים (הורים, מורים, ילדים) | □ | □ | □ |
| 21. | הוא חושב לפני שהוא עושה דברים | □ | □ | □ |
| 22. | הוא לוקח דברים שהם לא שלו מהבית, מבית ספר או ממקומות אחרים | □ | □ | □ |
| 23. | הוא מסתדר טוב יותר עם מבוגרים מאשר עם אנשים בגילו | □ | □ | □ |
| 24. | יש לו הרבה פחדים, הוא פוחד בקלות | □ | □ | □ |
| 25. | הוא מסיים את העבודה שהוא עושה, הקשב שלו טוב | □ | □ | □ |
